# Hepatoprotective Activity of Dried- and Fermented-Processed Virgin Coconut Oil

**DOI:** 10.1155/2011/142739

**Published:** 2011-01-26

**Authors:** Z. A. Zakaria, M. S. Rofiee, M. N. Somchit, A. Zuraini, M. R. Sulaiman, L. K. Teh, M. Z. Salleh, K. Long

**Affiliations:** ^1^Department of Biomedical Sciences, Faculty of Medicine and Health Science, Universiti Putra Malaysia, 43400 UPM Serdang, Selangor, Malaysia; ^2^Faculty of Pharmacy, Universiti Teknologi MARA, 40450 Shah Alam, Selangor, Malaysia; ^3^Pharmacogenomics Center, Faculty of Pharmacy, Universiti Teknologi MARA, 40450 Shah Alam, Selangor, Malaysia; ^4^Biotechnology Research Centre, Malaysian Agriculture Research Institute, 50744 Kuala Lumpur, Malaysia

## Abstract

The present study aims to determine the hepatoprotective effect of MARDI-produced virgin coconut oils, prepared by dried- or fermented-processed methods, using the paracetamol-induced liver damage in rats. Liver injury induced by 3 g/kg paracetamol increased the liver weight per 100 g bodyweight indicating liver damage. Histological observation also confirms liver damage indicated by the presence of inflammations and necrosis on the respective liver section. Interestingly, pretreatment of the rats with 10, but not 1 and 5, mL/kg of both VCOs significantly (*P* < .05) reduced the liver damage caused by the administration of paracetamol, which is further confirmed by the histological findings. In conclusion, VCO possessed hepatoprotective effect that requires further in-depth study.

## 1. Introduction

Liver is the largest organ in the human body and key organ of metabolism, including glycogen storage, decomposition of red blood cells, plasma protein synthesis, and detoxification [1]. It is continuously and variedly exposed to xenobiotics, environmental pollutants, and chemotherapeutic agents because of its strategic placement in the body [2]. If the natural protective mechanisms of the liver are overpowered during all such exposures, this will lead to hepatic injury. Liver diseases are worldwide problems, and conventional drugs used in the treatment of liver diseases are sometimes inadequate and can have serious adverse effects [3]. 

Despite remarkable strides in nowadays medicine field, modern drugs that directly or indirectly stimulate the liver function are hardly offering protection to this important organ from damage or aid in the regeneration of hepatic cells [4]. Those conventional drugs available for the treatment of liver disorders are at times inadequate and can have serious adverse effects [5]. There are a number of plant-based traditional medicines recommended for treatment of liver disorders to overcome the lack of reliable modern drugs with hepatoprotective effect [6]. Some of the herbs reportedly possess hepatoprotective effect including *Andrographis paniculata *[7], *Aquilegia vulgaris *[8],* Picrorhiza kurroa *[9], *Silybum marianum *[10], *Strychnos potatorum *[11], and* Tridax procumbens *[12]. 

Although there are a number of formulations containing herbal extracts employed in the traditional system of medicine sold in the market for liver disorders, especially in countries like India [13], China [14], and Malaysia [14], the management of liver disorders by herbal-based drugs or formulations is still considered to be an intriguing problem [4]. Although their biologically active components are unknown, herbal drugs are prescribed widely because of their effectiveness, fewer side effects, and relatively low cost [15]. Due to lack of awareness of a satisfactory remedy for serious liver diseases and increasing doubt on the efficacy and safety of the currently used drugs or herbal formulations, the quest to find effective and safe drugs or herbal medicines for liver disorders continues to be an area of interest. 

The health and nutritional benefits of coconut oil (CO) have been recognized for centuries, and CO has taken up an exceptional role in the diet as an important physiologically functional food [16]. Virgin coconut oil (VCO), known in Malaysia as “minyak kelapa dara”, is one type of coconut oil that has recently gain a lot of attention due to various claimed medicinal values, such as antioxidant [17], antimicrobial, antiviral [18–20], antihypercholesterol and antithrombotic [21] activities. Moreover, administration of VCO is capable of increasing antioxidant enzymes and reduces lipid peroxidation content [21]. According to Fife (2003), VCO is organic and produced through a low heat process from freshly harvested, hand-selected organically grown coconut. This cold process extraction conserves all of the functional components of coconuts (i.e., tocopherols, sterols, and squalene) and, at the same time, also maintained the structure of its fatty acid as no polymerization takes place. This accounts for the preservation of most of its natural antioxidants properties [16]. According to Van Immerseel et al. [20], lauric oil, a saturated carbon-12 medium chain fatty acid, encompasses the majority (48%–50%) of the nutritional content of VCO followed by a considerable amount of short-chain fatty acids such as capric, caproic, and caprylic acids. The link between antioxidant and hepatoprotective mechanisms has been established [4, 6] and has triggered the present study. In the present study, the objectives were to determine the hepatoprotective activity of dried- and fermented-produced VCOs in animal model.

## 2. Materials and Methods

### 2.1. Samples of VCOs

Dried- and fermented-processed VCOs, labelled as VCOA and VCOB, respectively, were provided by Dr. Kamariah Long from the Malaysian Agricultural Research and Development Institute (MARDI), Serdang, Selangor and stored at room temperature before used.

### 2.2. Preparation of VCOA

Preparation of VCOA was performed according to the methods described by Seow and Gwee [22] with several modifications. Briefly, coconut milk emulsion was centrifuged before chilling and thawing to allow better packing of the coconut oil globules. The temperature used were 10 and −4°C for chilling and freezing processes, respectively, while the thawing process was carried out in a water bath at 40°C until the coconut cream reached room temperature (25°C).

### 2.3. Preparation of VCOB

Preparation of VCOB was performed according to the methods described by Che Man et al. [23] with several modifications. Pure culture of *Lactobacillus plantarum* 1041 IAM was used to extract coconut oil. Grated coconut meat and water at 30°C was mixed in ratio of 1 : 1 and allowed to settle for 2 to 6 h. Coconut milk emulsion was then separated by adjusting pH of the coconut milk emulsion between pH 3 and 5.6.

### 2.4. Chemicals

Paracetamol (PCM; Sigma Chemicals, USA) was diluted in glycerol to the concentration of 3 g/kg. Silymarin (100 mg/kg; Sigma Aldrich, USA) was used as reference hepatoprotective agent and dissolved in distilled water.

### 2.5. Experimental Animal

Male *Sprague-Dawley* rats weighing between 180–220 g were used in the present study. The animals were obtained from the Veterinary Animal Unit, Universiti Putra Malaysia (UPM) and housed at the Animal House, Faculty of Medicine and Health Science, UPM. The animals were kept in polypropylene cages with wood shaving, fed with standard pellet and water *ad libitum* and maintained in a 12 h light/dark cycle at 27° ± 2°C. At all times, the rats were cared for in accordance with current UPM principles and guidelines for the care of laboratory animals and the UPM ethical guidelines for investigations of experimental pain in conscious animals as adopted from Zimmermann [24].

### 2.6. Acute Toxicity Studies

VCOA and VCOB, at the volume 10 mL/kg, were administered orally to normal rats. During 4 hours after the VCOs administration, the animals were observed for behavioural changes and mortality if any for 7 days.

### 2.7. PCM-Induced Hepatotoxicity in Rats

The PCM-induced hepatotoxicity model described by Dash et al. [13] was used with slight modifications. All animals were divided randomly into 9 groups of 6 rats each (*n* = 6). All groups were pre-treated orally either with 0.9% normal saline (NS), 100 mg/kg silymarin, VCOA, or VCOB at the different volumes (1, 5 and 10 mL/kg) for 7 consecutive days followed by single oral administration of 3 g/kg PCM or 100% glycerol (vehicle for diluting paracetamol). The administration of PCM or 100% glycerol was performed 24 hours after the last administration of both VCOs or silymarin. The animals were then anaesthetized after 48 h fasting following the last dose, and the blood were collected for biochemical parameters studies. Body weights of the rats were measured before and after treatment, and changes in body weights (as percentages) were recorded. The percentage of changes in body weights was calculated according to the following formula: *change in body weights *(*%*)* = *100* × *[(*Weight_n_* *−* *Weight_initial_*)*/Weight_initial_*].

### 2.8. Biochemical Studies of Blood Collected after Treatment with PCM

The blood (3 mL) was collected by cardiac puncture using sterile disposable syringe and kept in plain tube. The blood samples were allowed to clot for 45 min at room temperature. Serum was separated by centrifugation at 2500 rpm at 30°C for 15 min and utilized for the estimation of various biochemical parameters namely AST, AST, and ALP [25]. After collection of blood samples, the rats in different groups were sacrificed, their livers were excised immediately, and the liver weight/100 g body weight [26] was measured and fixed in 10% formalin for histopathology studies.

### 2.9. Histopathology Studies of Liver Collected after Treatment with PCM

Small pieces of liver tissues in each group were collected in 10% formalin for proper fixation. These tissues were processed and embedded in paraffin wax. Sections of 5 *μ*m in thickness were cut and stained with hematoxylin and eosin (H&E). These sections were examined under light microscope for histological changes and percentage of cell viability was calculated using the following formula: (%) cell viability = 100 × [(viable cell/(viable + death cell)] under a light microscope.

### 2.10. Statistical Analysis

The results are expressed as mean ± S.E.M. One-way analysis of variance (ANOVA) was applied for determining statistical significance of difference among the means calculated at the level of *P* < .05 using Turkey Post-Hoc test analysis.

## 3. Results

### 3.1. Acute Toxicity Studies of VCOs

No changes in behavioural and mortality were observed with oral administration of the highest volume (10 mL/kg) of VCOA or VCOB.

### 3.2. Effect of VCOA and VCOB on PCM-Induced Hepatotoxicity

#### 3.2.1. Effect of VCOA and VCOB on Body Weight

The effects of VCOA and VCOB on the body weight of PCM intoxicated rats are shown in Table 1. PCM significantly (*P* < .05) increased the rats body weight in group pre-treated with NS when compared to the group pretreated with NS followed by the vehicle. Interestingly, pretreatment with 10 mL/kg VCOA or VCOB, but not their 1 and 5 mL/kg concentrations, significantly (*P* < .05) reversed the PCM effect stated above and returned the rats body weight to normal value. One hundred mg/kg silymarin, on the other hand, also reduced the rats body weight to normal.

#### 3.2.2. Effect of VCOA and VCOB on Biochemical Parameter

The effects of VCOA and VCOB on serum level of hepatic enzymes, namely ALT, AST, and ALP, are shown in Table 2. PCM significantly (*P* < .05) increased the serum level of hepatic enzymes in group pre-treated with saline when compared to the group pretreated with NS followed by the vehicle. Interestingly, pretreatment with 10 mL/kg VCOA or VCOB, but not their 1 and 5 mL/kg concentrations, significantly (*P* < .05) reduced the serum level of ALT, AST, and ALP and reversed the PCM effect on the serum level of hepatic enzymes. In comparison, 100 mg/kg silymarin also reduced the serum level of hepatic enzymes to normal levels.

#### 3.2.3. Effect of VCOA and VCOB on Liver Weight

Administration of PCM following pretreatment with saline significantly (*P* < .05) induced a marked increase in liver weight per 100 g body weight when compared to the normal control group. Interestingly, pre-treatment with 10 mL/kg VCOA or VCOB, but not 1 and 5 mL/kg, significantly (*P* < .05) reversed the increase weight of liver seen in PCM-treated groups to the value obtained for normal control group (Table 3). Comparison with 100 mg/kg silymarin showed that the reference hepatoprotective agent did reduce the weight of liver to normal value.

#### 3.2.4. Histopathological Study of the Liver Pre-Treated with VCOA and VCOB Followed by Treatment with PCM

**Figure 1:**
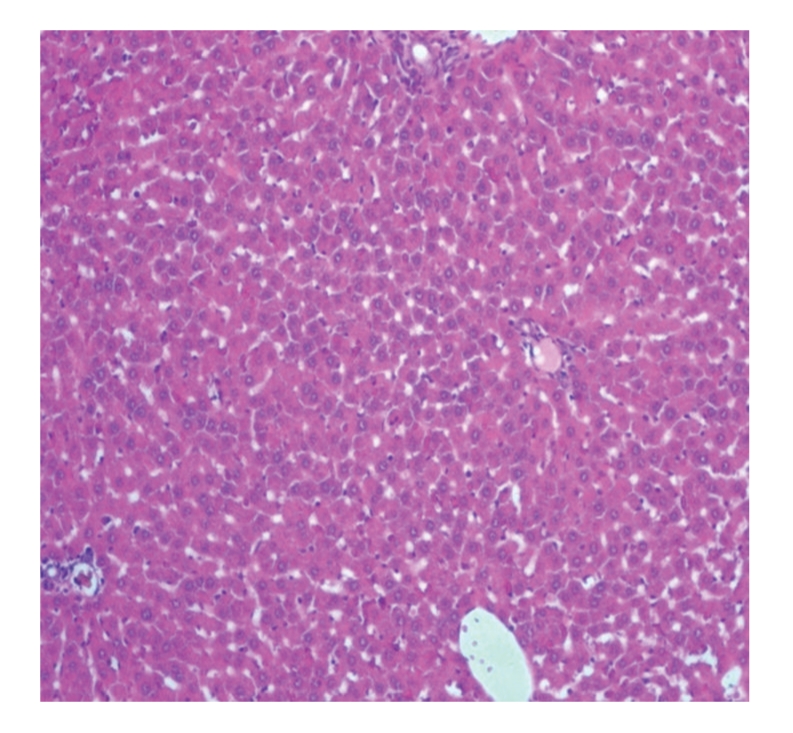
Section of the liver tissue of control group showing normal histology (40X). (Section of liver tissue of group pretreated with saline followed by 100% glycerol that exhibited normal histology is not shown.)

**Figure 2:**
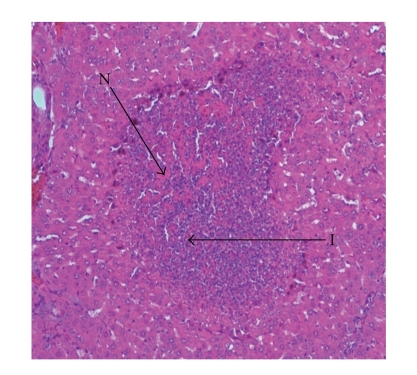
Section of the liver tissue of 3 g/kg PCM-treated group (p.o.) showing tissue necrosis (N) and inflammation (I) (40X).

**Figure 3:**
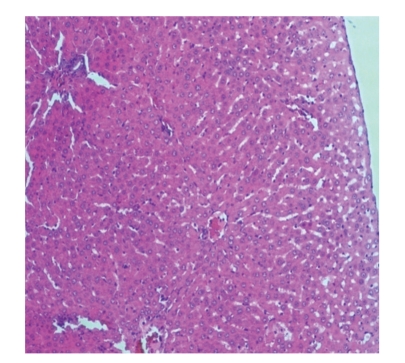
Section of the liver tissue of group pretreated with 10 mL/kg VCOA followed by PCM showing normal histology (40X).

**Figure 4:**
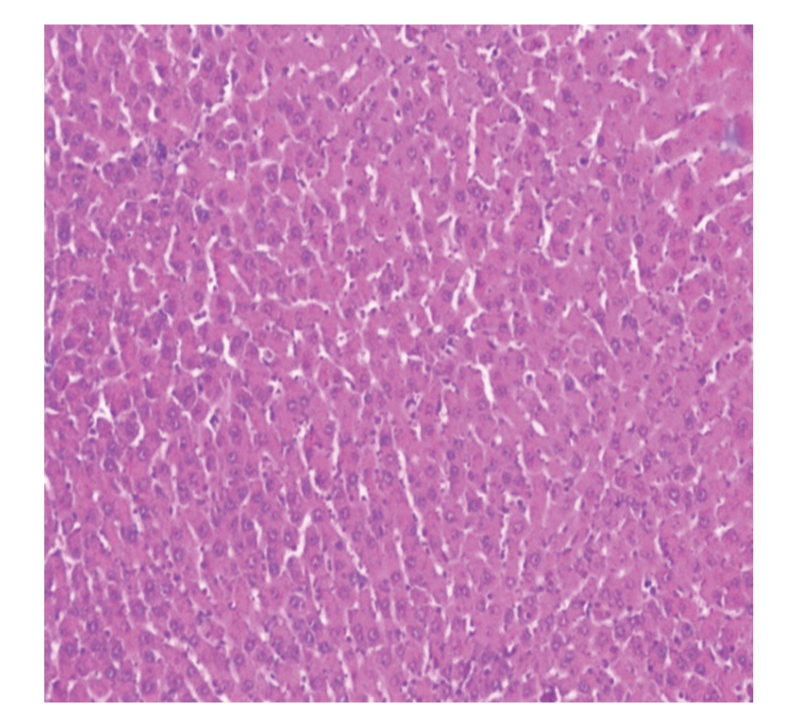
Section of the liver tissue of group pre-treated with 10 mL/kg VCOB followed by PCM showing normal histology (40X).

Liver sections from control rats pre-treated with normal saline followed by saline showed normal lobular architecture and normal hepatic cells with a well-preserved cytoplasm and well-defined nucleus and nucleoli (Figure 1). This normal architecture of hepatic cells was also seen in group pretreated with NS followed by 100% glycerol (figure not shown). Histopathological examination of the livers of rats pre-treated with 10 mL/kg VCOA or VCOB followed by the NS also demonstrated no significant morphological changes, as compared to the control group (figure not shown). Liver sections from animals pre-treated with NS followed by 3 g/kg PCM (toxin control group) showed marked regenerative activity in the form of binucleation, nuclear enlargement, and prominent nucleoli. Some cells showed loss of nucleus and nucleoli. The hepatoprotective effect of both VCOs was confirmed by the histopathological study (Figure 2). Interestingly, the liver sections of PCM-administered rats pre-treated with 10 mL/kg VCOA and VCOB (Figures 3 and 4) demonstrated normal liver histology indicated by the almost same architecture as the control group. However, the liver sections of PCM-administered rats pre-treated with 1 and 5 mL/kg VCOA or VCOB demonstrated almost the same architecture with the toxin control group in which signs of inflammation and necrosis were observed (figure not shown). In comparison, the liver tissue of rats pre-treated with reference hepatoprotective agent, 100 mg/kg silymarin also demonstrated normal liver histology (figure not shown).

#### 3.2.5. Effect of VCOA and VCOB on Viability of Cells

Table 4 shows the percentage of viable cells in rats treated with PCM following pre-treatment with VCOA or VCOB. Administration of PCM following the NS administration significantly (*P* < .05) decreased the percentage of viable cells compared to control group. Interestingly, only pre-treatment with 10 mL/kg VCOA and VCOB significantly (*P* < .05) reversed the effect of PCM on cell viability by maintaining the number of viable cells as seen with the control group. One hundred mg/kg silymarin, on the other hand, caused an increase in the rats liver cells' viability.

## 4. Discussion

Hepatic cells involve in various enzymatic metabolic activities, and damage to this organ will lead to disturbance in the body metabolism [27, 28]. PCM is mainly metabolised in liver to glucuronide and sulphate conjugates, which are easily excretable [29, 30]. Although PCM is safe in therapeutic doses, it can cause hepatic necrosis at toxic doses, which can be fatal in man and laboratory animals [31, 32], as a result of bioactivation to a toxic metabolite, N-acetyl-p-benzoquinone-imine (NAPQI) by cytochrome P450 monooxygenase [29, 30, 33]. The mechanism of hepatotoxicity of paracetamol has been studied extensively [34]. NAPQI toxicity occurs through its oxidative effects whereby it binds to macromolecules (proteins and DNA) and cellular proteins to produce protein adducts [25] and also by oxidizing lipids and altering homeostasis of calcium after depletion of glutathione [30]. These modified proteins caused the dysfunction and death of hepatocytes leading to liver necrosis [35].

Glutathione (GSH), the main intracellular nonprotein sulfhydryl, plays an important role in the maintenance of cellular proteins and lipids in their functional states wherein the sulfhydryl compounds are known to be among the most important endogenous antioxidants. NAPQI binding to GSH leads to the formation of a conjugate which results in oxidation and conversion of GSH to GSSG (oxidized form of glutathione) [36]. This process lowered the GSH level and exacerbated the toxic effects of oxidative insult, resulting in increased membrane and cell damage. Other protein and nonprotein sulfhydryl groups found in the cell will take over at this moment to provide an important alternate protection [37].

Protection against PCM-induced toxicity has been generally used as a test for screening the potential hepatoprotective activity of extracts/compounds [32, 38, 39]. The evidences of PCM-induced liver injury include increase in liver weight [40] and elevation on serum level of hepatic enzymes [41], which is due to leakage of cellular enzymes (ALP, AST, and ALT) into plasma [32]. When the plasma membrane of liver cell is damaged, various enzymes normally found in the cytosol are released into blood stream. Estimation of these enzymes level in the serum has been considered as a useful quantitative marker to describe the extent and type of hepatocellular damage [32, 42]. In the present study, PCM, at the toxic dose used and as expected, increased the liver weight and elevated the serum levels of the respective hepatic enzymes [30]. In addition to these, PCM also increased the rats body weight and reduced the number of viable cells and this is further supported by histological studies that showed marked sign of inflammation and gross necrosis of the centrilobular hepatocytes characterised by nuclear pyknosis, karyolysis, and eosinophilic infiltration in liver pre-treated with PCM alone.

Based on the pathophysiology processes involved in the PCM-induced hepatotoxicity, it is believed that compound/extract with antioxidant activity would be a good hepatoprotective candidate. In addition, compounds/extracts possessing immunomodulatory and anti-inflammatory properties have been suggested to possess antioxidant properties [43]. In concurrence with all these claims, both VCOA and VCOB have been reported to contain polyphenolic compounds and to possess antioxidant activity [44–47] and to exhibit antiulcerogenic (Malarvili et al., 2010; personal comm.), antinociceptive, and anti-inflammatory activities [48]. The present study, for the first time, revealed the potential of VCOs produced either via the dried- or fermented-process to demonstrate hepatoprotective activity in rats against paracetamol-induced liver injury. The hepatoprotective activity, which is seen at the highest concentration (10 mL/kg) of VCOs used, was also accompanied by the VCOs ability to reduce PCM-induced increase in body and liver weights to normal values, and to reduce the serum level of ALT, AST, and ALP increased after pre-treatment with PCM. The serum level of hepatic enzymes, namely AST, ALT, and ALP, is being used clinically and experimentally as a routine procedure to assess and monitor the functional status of the liver [36]. These enzymes are used as serum markers of hepatic damage and elevated levels of these enzymes in serum as seen in PCM-treated group indicating liver dysfunction. Interestingly, pretreatment with VCOA or VCOB caused significant reduction to these elevations, demonstrating that both VCOs also possessed capability to maintain the functional capacity of the liver. The raised serum liver enzymes in intoxicated rats can be attributed to the damage in the histostructural integrity of the hepatocytes [25]. The VCOs used in the present study protected the structural integrity of hepatocyte membrane as evident from the hepatoprotection provided by the oils, which in turn, lead to inhibition of the increase in serum liver enzymes. 

This beneficial effect of VCOA and VCOB on biochemical parameters is further supported by histopathological observations. The regenerative activity observed in liver cells of PCM-treated animals indicates the compensatory changes as a result of cellular insult. The preserved lobular architecture and less prominent regenerative activity seen in liver sections of animals pretreated with VCOA or VCOB suggested that both VCOs help to retain the structural integrity of liver against PCM-induced toxicity. Interestingly, the recovery towards normalization of serum enzymes and liver histological architecture caused by VCOA and VCOB was almost similar to that caused by silymarin [49]. Silymarin is a known hepatoprotective compound that has been reported to possess a protective effect on the plasma membrane of hepatocytes [49, 50]. In addition, both VCOs also successfully increased the number of viable cells reduced after pre-treatment with PCM while histological studies of liver pre-treated with VCOA or VCOB followed by PCM demonstrated the capability of both VCOs to preserve the structural integrity of the hepatocellular membrane as seen with normal untreated cells with minimal necrosis in centrilobular and regeneration of hepatocytes seen. These findings concur with the ability of both VCOs to reduce the serum enzymes level as compared to the enzyme level in the hepatotoxin-treated rats and may be attributed to the oils inhibitory effects on cytochrome P450 or/and promotion of its glucuronidation [28, 51, 52]. Other possible mechanisms by which those VCOs exert their protective action against PCM-induced hepatocellular metabolic alterations include stimulation of hepatic regeneration via an enhanced synthesis of protein and glycoprotein or accelerated detoxification and excretion [32], stabilization of the hepatocellular membrane, and prevention of the process of lipid peroxidation [49]. However, further studies are warranted before we could conclude on the exact mechanism(s) involved in the hepatoprotective activity of the VCOs. However, the presence of polyphenols and the antioxidant activity demonstrated by both VCOs as reported by our colleagues are suggested to contribute partly to the observed hepatoprotective activity of VCOs [4]. Furthermore, there is also a claim that the combination of hepatoprotective effect and antioxidant activity synergistically prevents the process of initiation and progress of hepatocellular damage [53].

Despite a promising hepatoprotective activity exhibited by both VCOs, the activity was observed only at the highest concentration (10 mL/kg). The failure of both VCOs to protect the liver from PCM-induced toxicity at their lowest concentrations (1 and 5 mL/kg) could be related to the oils fatty acids content. More than 50% of the fatty acids found in both VCOs consist of medium chain fatty acids (MCFAs), which include C_6_ to C_12_ fatty acids (e.g., caproic acid, caprylic acid, capric acid, and lauric acid) [48]. The smaller medium-chain triglyceride molecules found in coconut oil are easily digested and absorbable. The digested MCTs will form free MCFAs and monoglycerides, which will enter the mucosal cell of the intestines and be carried to the liver without further metabolism. This ensures maximal exposure to the hepatic metabolic enzymes. MCFA will be metabolized rapidly in the liver mitochondria via oxidation process to ketone bodies, CO_2_, and energy. However, the other MCFAs and monoglyceride molecules that are not metabolized in the mitochondria will enter the systemic circulation to exert their systemic effects and action [54]. It is, thus, suggested that both VCOs exhibit hepatoprotective effect only at their higher concentration (10 mL/kg) because only at this concentration a significant amount of MCFAs and monoglyceride molecules could escape from being metabolized by the liver enzymes and exert their effects systemically. Other than that, it is believed that only at higher concentration both VCOs contained significant amount of polyphenols that could help protect the liver from PCM toxicity.

Based on our present findings, it can be concluded that VCOs, regardless of the differences in their methods of preparation, possess a promising hepatoprotective effect and this hepatoprotective effect of VCO may be attributed, partly to its antioxidant activity. Further studies are needed before the exact conclusion could be drawn on the actual mechanisms of hepatoprotective involved. This report may serve as a starting point for further and extensive studies on the pharmacological potential of VCOs.

## Figures and Tables

**Figure 5:**
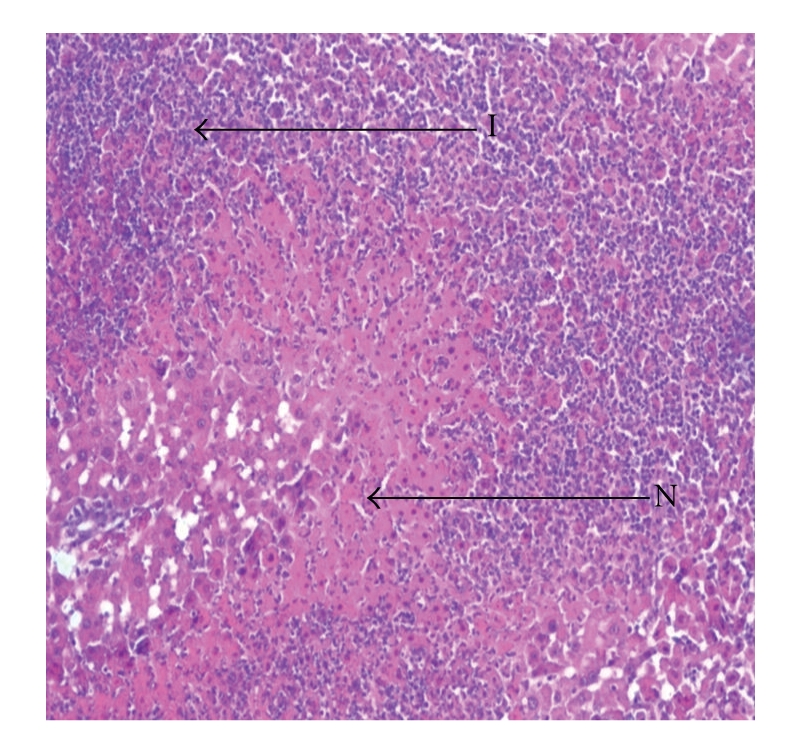
Section of the liver tissue of group pre-treated with 5 mL/kg VCOA followed by PCM showing tissue necrosis (N) and inflammation (I) (40X). (Section of the liver tissues pretreated with 1 mL/kg VCOA or VCOB followed by PCM is not shown.)

**Figure 6:**
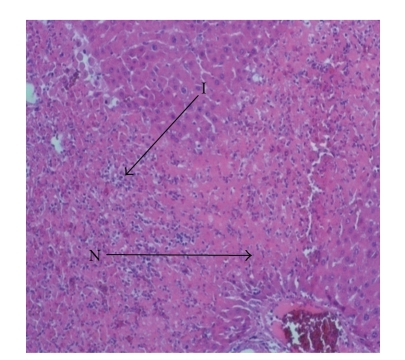
Section of the liver tissue of group pre-treated with 5 mL/kg VCOB followed by PCM showing tissue necrosis (N) and inflammation (I) (40X).

**Table 1:** Effects of VCOA and VCOB on the percentage change of body weight in PCM-treated rats.

Group	Change in body weight (%)
NS + Vehicle	3.54 ± 0.94^b^
NS + PCM	11.17 ± 0.53^a^
Silymarin + PCM	3.87 ± 0.61^b^

1 mL/kg VCOA + PCM	13.41 ± 2.07^a^
5 mL/kg VCOA + PCM	13.70 ± 2.18^a^
10 mL/kg VCOA + PCM	2.55 ± 0.93^b^

1 mL/kg VCOB + PCM	13.30 ± 0.95^a^
5 mL/kg VCOB + PCM	14.58 ± 1.19^a^
10 mL/kg VCOB + PCM	5.51 ± 0.57^b^

Value are mean ± S.E.M. Mean with different letters differ significantly (*P* < .05).

**Table 2:** Effects of VCOA and VCOB on the hepatic enzymes levels in serum of PCM-treated rats.

Group	Hepatic enzyme Level (U/L)		
	ALT	AST	ALP
NS + Vehicle	2.03 ± 0.77^a^	2.75 ± 0.19^a^	162.27 ± 2.63^a^
NS + PCM	18.88 ± 1.29^b^	22.50 ± 3.86^b^	311.55 ± 14.79^b^
Silymarin + PCM	4.21 ± 0.92^a^	3.84 ± 0.73^a^	177.41 ± 10.85^a^

1 mL/kg VCOA + PCM	14.73 ± 2.65^b^	17.50 ± 0.35^b^	291.45 ± 17.52^b^
5 mL/kg VCOA + PCM	16.48 ± 1.99^b^	17.76 ± 0.96^b^	295.38 ± 17.76^b^
10 mL/kg VCOA + PCM	7.90 ± 0.71^c^	6.97 ± 0.96^c^	192.23 ± 8.88^a^

1 mL/kg VCOB + PCM	14.68 ± 0.94^b^	18.46 ± 3.73^b^	295.70 ± 15.73^b^
5 mL/kg VCOB + PCM	13.91 ± 1.02^b^	19.20 ± 2.03^b^	295.56 ± 35.62^b^
10 mL/kg VCOB + PCM	7.73 ± 0.83^c^	8.06 ± 0.17^c^	189.28 ± 8.85^a^

Values are mean ± S.E.M. Means with different letters differ significantly (*P* < .05).

**Table 3:** Effects of VCOA and VCOB on the liver weight of PCM-treated rats.

Group	Liver weight (g/100g body weight)
NS + Vehicle	3.50 ± 0.12^a^
NS + PCM	4.38 ± 0.17^b^

Silymarin + PCM	3.76 ± 0.21^a^

1 mL/kg VCOA + PCM	4.50 ± 0.12^b^
5 mL/kg VCOA + PCM	4.47 ± 0.13^b^
10 mL/kg VCOA + PCM	3.30 ± 0.19^a^

1 mL/kg VCOB + PCM	4.60 ± 1.64^b^
5 mL/kg VCOB + PCM	4.73 ± 0.26^b^
10 mL/kg VCOB + PCM	3.51 ± 0.17^a^

Value are mean ± S.E.M. Mean with different letters differ significantly (*P* < .05).

**Table 4:** Effects of VCOA and VCOB on the percentage of viable cells in PCM-treated rats.

Group	Percentage of viable cells (%)
NS + Vehicle	90.00 ± 0.98^a^
NS + PCM	9.00 ± 0.38^b^
Silymarin + PCM	93.13 ± 1.22^a^
1 mL/kg VCOA + PCM	11.68 ± 0.40^b^
5 mL/kg VCOA + PCM	11.75 ± 0.43^b^
10 mL/kg VCOA + PCM	91.16 ± 0.67^a^
1 mL/kg VCOB + PCM	11.31 ± 0.54^b^
5 mL/kg VCOB + PCM	11.19 ± 0.27^b^
10 mL/kg VCOB + PCM	91.60 ± 1.79^a^

Values are mean ± S.E.M. Means with different letters differ significantly (*P* < .05).

## References

[1] Opoku AR, Ndlovu IM, Terblanche SE, Hutchings AH (2007). In vivo hepatoprotective effects of *Rhoicissus tridentata* subsp. *cuneifolia*, a traditional Zulu medicinal plant, against CCl_4_-induced acute liver injury in rats. *South African Journal of Botany*.

[2] Ibrahim M, Khaja MN, Aara A (2008). Hepatoprotective activity of *Sapindus mukorossi* and *Rheum emodi* extracts: in vitro and in vivo studies. *World Journal of Gastroenterology*.

[3] Arhoghro EM, Ekpo KE, Anosike EO, Ibeh GO (2009). Effect of aqueous extract of bitter leaf (*Vernonia Amygdalina* Del) on carbon tetrachloride (CCl_4_) induced liver damage in albino wistar rats. *European Journal of Scientific Research*.

[4] Chattopadhyay RR (2003). Possible mechanism of hepatoprotective activity of *Azadirachta indica* leaf extract: part II. *Journal of Ethnopharmacology*.

[5] Öbek H, Uğraş S, Bayram I (2004). Hepatoprotective effect of *Foeniculum vulgare* essential oil: a carbon-tetrachloride induced liver fibrosis model in rats. *Scandinavian Journal of Laboratory Animal Science*.

[6] Pramyothin P, Ngamtin C, Poungshompoo S, Chaichantipyuth C (2007). Hepatoprotective activity of *Phyllanthus amarus* Schum. et. Thonn. extract in ethanol treated rats: in vitro and in vivo studies. *Journal of Ethnopharmacology*.

[7] Pramyothin P, Udomuksorn W, Poungshompoo S, Chaichantipyuth C (1994). Hepatoprotective effect of *Andrographis paniculata* and its constituent, andrographolide, on ethanol hepatotoxicity in rats. *Asia Pacific Journal of Pharmacology*.

[8] Jodynis-Liebert J, Matławska I, Bylka W, Murias M (2005). Protective effect of *Aquilegia vulgaris* (L.) on APAP-induced oxidative stress in rats. *Journal of Ethnopharmacology*.

[9] Saraswat B, Visen PKS, Patnaik GK, Dhawan BN (1999). Ex vivo and in vivo investigations of picroliv from *Picrorhiza kurroa* in an alcohol intoxication model in rats. *Journal of Ethnopharmacology*.

[10] Flora K, Hahn M, Rosen H, Benner K (1998). Milk thistle (*Silybum marianum*) for the therapy of liver disease. *American Journal of Gastroenterology*.

[11] Sanmugapriya E, Venkataraman S (2006). Studies on hepatoprotective and antioxidant actions of *Strychnos potatorum* Linn. seeds on CCl_4_-induced acute hepatic injury in experimental rats. *Journal of Ethnopharmacology*.

[12] Ravikumar V, Shivashangari KS, Devaki T (2006). Hepatoprotective activity of *Tridax procumbens* against D-galactosamine/lipopolysaccharide-induced hepatitis in rats. *Journal of Ethnopharmacology*.

[13] Dash DK, Yeligar VC, Nayak SS (2007). Evaluation of hepatoprotective and antioxidant activity of *Ichnocarpus frutescens* (Linn.) R.Br. on paracetamol-induced hepatotoxicity in rats. *Tropical Journal of Pharmaceutical Research*.

[14] Sadasivan S, Latha PG, Sasikumar JM, Rajashekaran S, Shyamal S, Shine VJ (2006). Hepatoprotective studies on *Hedyotis corymbosa* (L.) Lam. *Journal of Ethnopharmacology*.

[15] Valiathan MS (1998). Healing plants. *Current Science*.

[16] Fife B (2003). *The Healing Miracles of Coconut Oil*.

[17] Santhosh S, Anandan R, Sini TK, Mathew PT, Thankappan TK (2005). Biochemical studies on the antiulcer effect of glucosamine on antioxidant defense status in experimentally induced peptic ulcer in rats. *Journal of Clinical Biochemistry and Nutrition*.

[18] Bergsson G, Arnfinnsson J, Karlsson SM, Steingri Msson O, Thormar H (1998). In vitro inactivation of Chlamydia trachomatis by fatty acids and monoglycerides. *Antimicrobial Agents and Chemotherapy*.

[19] German JB, Dillard CJ (2004). Saturated fats: what dietary intake?. *American Journal of Clinical Nutrition*.

[20] Van Immerseel F, De Buck J, Boyen F (2004). Medium-chain fatty acids decrease colonization and invasion through hilA suppression shortly after infection of chickens with *Salmonella enterica* serovar enteritidis. *Applied and Environmental Microbiology*.

[21] Nevin KG, Rajamohan T (2004). Beneficial effects of virgin coconut oil on lipid parameters and in vitro LDL oxidation. *Clinical Biochemistry*.

[22] Seow CC, Gwee CN (1997). Coconut milk: chemistry and technology. *International Journal of Food Science and Technology*.

[23] Che Man YB, Abdul Karim MIB, Teng CT (1997). Extraction of coconut oil with *Lactobacillus plantarum* 1041 IAM. *Journal of the American Oil Chemists’ Society*.

[24] Zimmermann M (1983). Ethical guidelines for investigations of experimental pain in conscious animals. *Pain*.

[25] Somchit MN, Zuraini A, Ahmad Bustaman A, Somchit N, Sulaiman MR, Noratunlina R (2005). Protective activity of turmeric (*Curcuma longa*) in paracetamol-induced hepatotoxicity in rats. *International Journal of Pharmacology*.

[26] Somchit N, Wong CW, Zuraini A (2006). Involvement of phenobarbital and SKF 525A in the hepatotoxicity of antifungal drugs itraconazole and fluconazole in rats. *Drug and Chemical Toxicology*.

[27] Melmon KL, Morrelli HF, Hoffman BB, Nierenberg DW (1992). *Melmon and Morrelli’s Clinical Pharmacology: Basic Principles of Therapeutics*.

[28] Porchezhian E, Ansari SH (2005). Hepatoprotective activity of *Abutilon indicum* on experimental liver damage in rats. *Phytomedicine*.

[29] Katzung BG (2004). *Basic and Clinical Pharmacology*.

[30] Jafri MA, Subhani MJ, Javed K, Singh S (1999). Hepatoprotective activity of leaves of *Cassia occidentalis* against paracetamol and ethyl alcohol intoxication in rats. *Journal of Ethnopharmacology*.

[31] Eriksson LS, Broome U, Kalin M, Lindholm M (1992). Hepatotoxicity due to repeated intake of low doses of paracetamol. *Journal of Internal Medicine*.

[32] Kumar G, Banu GS, Pappa PV, Sundararajan M, Pandian MR (2004). Hepatoprotective activity of *Trianthema portulacastrum* L. against paracetamol and thioacetamide intoxication in albino rats. *Journal of Ethnopharmacology*.

[33] Dahlin DC, Miwa GT, Lu AY, Nelson SD (1984). N-acetyl-p-benzoquinone imine: a cytochrome P-450-mediated oxidation product of acetaminophen. *Proceedings of the National Academy of Sciences of the United States of America*.

[34] Hazai E, Vereczkey L, Monostory K (2002). Reduction of toxic metabolite formation of acetaminophen. *Biochemical and Biophysical Research Communications*.

[35] Park BK, Pirmohamed M, Kitteringham NR (1992). Idiosyncratic drug reactions: a mechanistic evaluation of risk factors. *British Journal of Clinical Pharmacology*.

[36] Yanpallewar SU, Sen S, Tapas S, Kumar M, Raju SS, Acharya SB (2002). Effect of *Azadirachta indica* on paracetamol-induced hepatic damage in albino rats. *Phytomedicine*.

[37] Genet S, Kale RK, Baquer NZ (2000). Effects of free radicals on cytosolic creatine kinase activities and protection by antioxidant enzymes and sulfhydryl compounds. *Molecular and Cellular Biochemistry*.

[38] Singh A, Handa SS (1995). Hepatoprotective activity of *Apium graveolens* and *Hygrophila auriculata* against paracetamol and thioacetamide intoxication in rats. *Journal of Ethnopharmacology*.

[39] Ahmed MB, Khater MR (2001). Evaluation of the protective potential of *Ambrosia maritima* extract on acetaminophen-induced liver damage. *Journal of Ethnopharmacology*.

[40] Rasheed RA, Ali BH, Bashir AK (1995). Effect of *Teucrium stocksianum* on paracetamol-induced hepatotoxicity in mice. *General Pharmacology*.

[41] Kaplowitz N (2001). Drug-induced liver disorders: implications for drug development and regulation. *Drug Safety*.

[42] Ansari RA, Tripathi SC, Patnaik GK, Dhawan BN (1991). Antihepatotoxic properties of picroliv: an active fraction from rhizomes of *Picrorhiza kurrooa*. *Journal of Ethnopharmacology*.

[43] Devasagayam TPA, Sainis KB (2002). Immune system and antioxidants, especially those derived from Indian medicinal plants. *Indian Journal of Experimental Biology*.

[44] Sellappan S, Akoh CC, Krewer G (2002). Phenolic compounds and antioxidant capacity of Georgia-grown blueberries and blackberries. *Journal of Agricultural and Food Chemistry*.

[45] Nevin KG, Rajamohan T (2008). Influence of virgin coconut oil on blood coagulation factors, lipid levels and LDL oxidation in cholesterol fed Sprague-Dawley rats. *European e-Journal of Clinical Nutrition and Metabolism*.

[46] Nevin KG, Rajamohan T (2006). Virgin coconut oil supplemented diet increases the antioxidant status in rats. *Food Chemistry*.

[47] Marina AM, Che Man YB, Nazimah SAH, Amin I (2009). Chemical properties of virgin coconut oil. *Journal of the American Oil Chemists’ Society*.

[48] Zakaria ZA, Ahmad Z, Somchit MN (2010). Antihypercholesterolemia property and fatty acid composition of mardi-produced virgin coconut oils. *African Journal of Pharmacy and Pharmacology*.

[49] Mujeeb M, Aeri V, Bagri P, Khan S (2009). Hepatoprotective activity of the methanolic extract of *Tylophora indica* (Burm. f.) Merill. leaves. *International Journal of Green Pharmacy*.

[50] Ramellini G, Meldolesi J (1976). Liver protection by silymarin: in vitro effect on dissociated rat hepatocytes. *Arzneimittel-Forschung*.

[51] Clark WG, Craig Brater D, Johnson AR (1992). *Goth's Medical Pharmacology*.

[52] Gilman AG, Rall TW, Nies AS, Taylor P (1990). *Goodman and Gilman’s The Pharmacological Basis of Therapeutics*.

[53] Gupta AK, Chitme H, Dass SK, Misra N (2010). Hepatoprotective activity of *Rauwolfia serpentina* rhizome in paracetamol intoxicated rats. *Journal of Pharmacology and Toxicology*.

[54] Dayrit CS (2005). *Truth about Coconut Oil—The Drugstore in a Bottle*.

